# The γ-Aminobutyric Acid (GABA) Alleviates Salt Stress Damage during Seeds Germination of White Clover Associated with Na^+^/K^+^ Transportation, Dehydrins Accumulation, and Stress-Related Genes Expression in White Clover

**DOI:** 10.3390/ijms19092520

**Published:** 2018-08-25

**Authors:** Bizhen Cheng, Zhou Li, Linlin Liang, Yiqin Cao, Weihang Zeng, Xinquan Zhang, Xiao Ma, Linkai Huang, Gang Nie, Wei Liu, Yan Peng

**Affiliations:** Department of Grassland Science, College of Animal Science and Technology, Sichuan Agricultural University, Chengdu 611130, China; Chengbizhengrass@163.com (B.C.); lizhou1986814@163.com (Z.L.); LiangllCLJ@163.com (L.L.); c545755756@163.com (Y.C.); zengwh0123@163.com (W.Z.); zhangxq@sicau.edu.cn (X.Z.); maroar@126.com (X.M.); huanglinkai@sicau.edu.cn (L.H.); nieganggrass@hotmail.com (G.N.); lwgrass@126.com (W.L.)

**Keywords:** growth, osmotic adjustment, metabolism, dehydrins, antioxidant, transcript

## Abstract

The objective of this study was to determine the effect of soaking with γ-aminobutyric acid (GABA) on white clover (*Trifolium repens* cv. Haifa) seed germination under salt stress induced by 100 mM NaCl. Seeds soaking with GABA (1 μM) significantly alleviated salt-induced decreases in endogenous GABA content, germination percentage, germination vigor, germination index, shoot and root length, fresh and dry weight, and root activity of seedling during seven days of germination. Exogenous application of GABA accelerated starch catabolism via the activation of amylase and also significantly reduced water-soluble carbohydrate, free amino acid, and free proline content in seedlings under salt stress. In addition, improved antioxidant enzyme activities (SOD, GPOX, CAT, APX, DHAR, GR and MDHR) and gene transcript levels (*Cu/ZnSOD*, *FeSOD, MnSOD*, *CAT*, *GPOX*, *APX*, *MDHR*, *GPX* and *GST*) was induced by seeds soaking with GABA, followed by decreases in O_2_^∙−^, H_2_O_2_, and MDA accumulation during germination under salt stress. Seeds soaking with GABA could also significantly improve Na^+^/K^+^ content and transcript levels of genes encoding Na^+^/K^+^ transportation (*HKT1*, *HKT8*, *HAL2*, *H^+^-ATPase* and *SOS1*) in seedlings of white clover. Moreover, exogenous GABA significantly induced the accumulation of dehydrins and expression of genes encoding dehydrins (*SK2, Y2K, Y2SK*, and *dehydrin b*) in seedlings under salt stress. These results indicate that GABA mitigates the salt damage during seeds germination through enhancing starch catabolism and the utilization of sugar and amino acids for the maintenance of growth, improving the antioxidant defense for the alleviation of oxidative damage, increasing Na^+^/K^+^ transportation for the osmotic adjustment, and promoting dehydrins accumulation for antioxidant and osmotic adjustment under salt stress.

## 1. Introduction

It has been reported that over 900 million hectares of land is negatively affected by salinity, accounting for 7% of the world’s total land [1]. Salinity has become a major problem limiting the growth and development of various plants. Effects of salt damage on plants are involved in multiple morphological, physiological, and biochemical processes [2]. For example, plant roots could be negatively affected by high concentration of Na^+^ and Cl^−^. The overaccumulation of Na^+^ and Cl^−^ in plants causes ion toxicity and also decreases the absorption of other ions leading to growth inhibition and metabolic disturbance under salt stress. In addition, salt stress seriously reduces the ability of plants taking up water resulting in physiological drought [3,4,5,6]. Those negative effects directly or indirectly lead to the overaccumulation of reactive oxygen species (ROS) which cause oxidative damage in plants under salt stress [7]. It is well-known that salt stress negatively affects all vegetative stages of plants, especially for seed germination [8]. Seed germination is the most important and sensitive process during plant growth and development. Previous studies have indicated that salt stress significantly reduced germination percentage, germination vigor, shoot and root length of different plant species [9,10,11].

The γ-aminobutyric acid (GABA) is a natural nonprotein amino acid that exists in both animals and plants. GABA has been widely studied as an important inhibitory neurotransmitter in animals [12]. In plants, GABA is associated with the maintenance of the carbon-nitrogen balance, the metabolism of amino acids and carbohydrates, and the regulation of growth and cell pH [13]. GABA is also involved in plants response to abiotic stresses, diseases, and insects [14,15,16]. Some studies have shown that the accumulation of GABA rapidly increased when plants were exposed to many adverse conditions, such as hypoxia, drought, cold, high temperature, low light, and high salt, indicating GABA could play a major role during abiotic stresses [14]. It has been reported that the GABA in plant cells could act as an effective osmolyte without toxic effects during the salt-induced dehydration and had the function of ROS scavenging under stressful environmental condition [17]. In addition, it has been found that the suitable concentration of exogenous GABA could promote plant growth, antioxidant metabolism, and transcript levels of genes encoding antioxidant enzymes, thereby alleviating stress-caused oxidative damage in plants [14,15]. Exogenous application of GABA could also regulate the osmotic balance in plant cells contributing to the enhancement of stress tolerance [18,19,20]. More importantly, exogenous GABA effectively inhibited the production of H_2_O_2_ and reduced oxidative damage through regulating the expression of key genes of H_2_O_2_ production and genes encoding peroxidases in *Caragana* *intermedia* roots under salt stress [14]. Seed soaking with GABA also significantly increased seed germination percentage and reduced the salt-caused injury during seeds germination of wheat and maize [21], but the possible mechanism still needs to be further investigated.

As an important legume forage with high content of crude protein, white clover (*Trifolium* *repens* L.) is widely cultivated all over the world, but it is susceptible to salt stress [22,23]. As mentioned above, previous studies have proved that exogenous GABA could significantly improve the tolerance to abiotic stresses in various plant species, but little information is available about GABA-regulated salt tolerance during seeds germination. The purposes of this study were (1) to examine effects of seeds soaking with the GABA on germination characteristics and (2) to reveal GABA-regulated salt tolerance associated with antioxidant defense, osmotic adjustment, Na^+^/K^+^ transportation, dehydrins accumulation, and relevant genes expression during seeds germination of white clover under salt stress. The study will largely contribute to further understanding salt-tolerant mechanism induced by GABA in plants.

## 2. Results

### 2.1. Effects of the GABA on Seed Germination Characteristics

The germination vigor (GV), germination percentage (GP), germination index (GI), mean germination time (MGT), and seed vigour index (VI) significantly decreased in response to salt stress. Exogenous GABA did not have significant effects on GV, GP, GI, MGT, and VI under the normal water condition. The low concentration (0.5 and 1 μM) of GABA significantly improved GV, GP, GI, and VI, whereas high concentration (5 mM) of GABA inhibited seeds germination of white clover under salt stress (Table 1). Under salt stress, seeds primed with 0.5 and 1 μM GABA had 6.5% and 11.77% higher GP than seeds primed with water, respectively (Table 1). Under normal water condition, seeds soaking with the different concentration of GABA did not have significant effects on seedlings fresh weight (FW), dry weight (DW), root length (RL), shoot length (SL), and shoot-root ratio (Table 2). Under salt stress, seedling FW, DW, RL, SL and shoot-root ratio significantly decreased. Seeds priming with exogenous GABA (1 μM) had significantly higher seedlings DW, longer SL, and shoot-root ratio than seeds priming with water in response to salt stress (Table 2). Similarly, seeds soaking with lower concentration of GABA (0.5, 1, 2.5 μM) maintained significantly higher seedlings FW than seeds priming with water under salt stress (Table 2).

### 2.2. Effect of the GABA on Root Activity and Endogenous GABA Content

**Figure 1:**
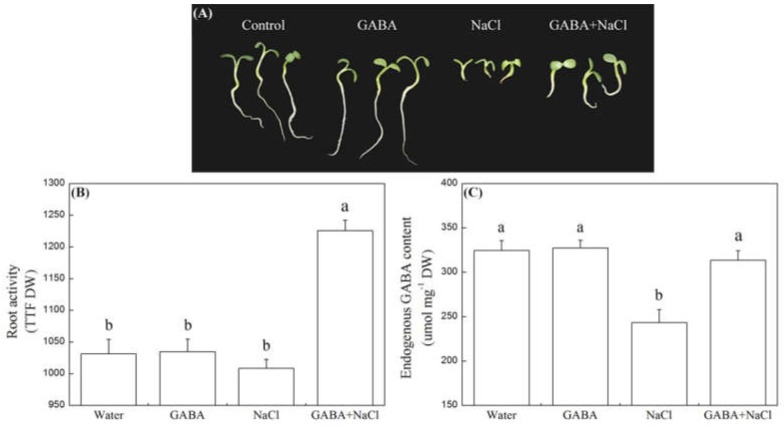
Effects of seed soaking with GABA or water on (**A**) phenotypic changes, (**B**) root activity, and (**C**) endogenous GABA content during seeds germination (7 days) under salt stress. Vertical bars indicate ± SE of mean (*n* = 6). Different letters above indicate significant difference. LSD (*p* ≤ 0.05).

Figure 1A showed the phenotypic difference between seeds soaking with GABA and water after seven days of germination under normal and salt stress conditions. The root activity and endogenous GABA content of seedlings between seeds soaking with GABA and water did not show significant difference under normal water condition, but seeds soaking with GABA exhibited significantly higher root activity than seeds soaking with water under salt stress. The root activity and endogenous GABA content of seedlings with GABA treatment increased by 21.6 and 62.2% compared to the seedlings without GABA treatment under salt stress, respectively (Figure 1B,C).

### 2.3. Effects of the GABA on Starch Metabolism and Osmotic Adjustment

**Figure 2:**
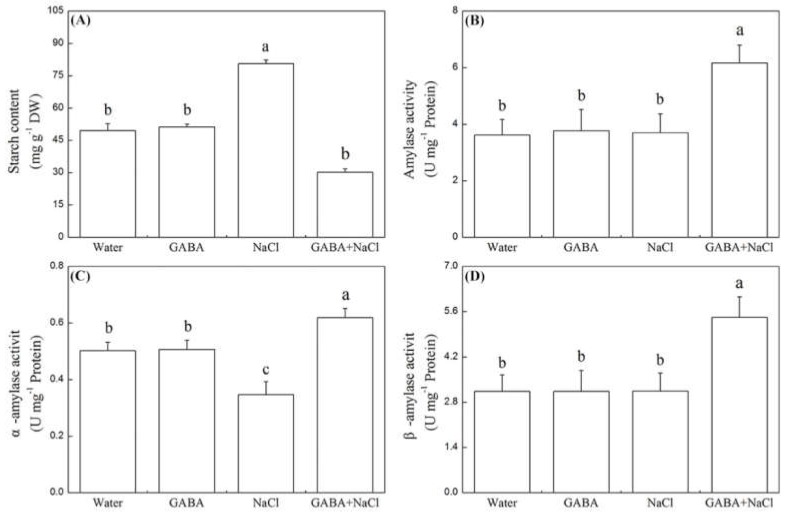
Effects of seed soaking with GABA or water on (**A**) starch content, (**B**) amylase activity, (**C**) α-amylase activity, and (**D**) β-amylase activity during seeds germination (7 days) under salt stress. Vertical bars indicate ± SE of mean (n = 6). Different letters above indicate significant difference. LSD (*p* ≤ 0.05).

**Figure 3:**
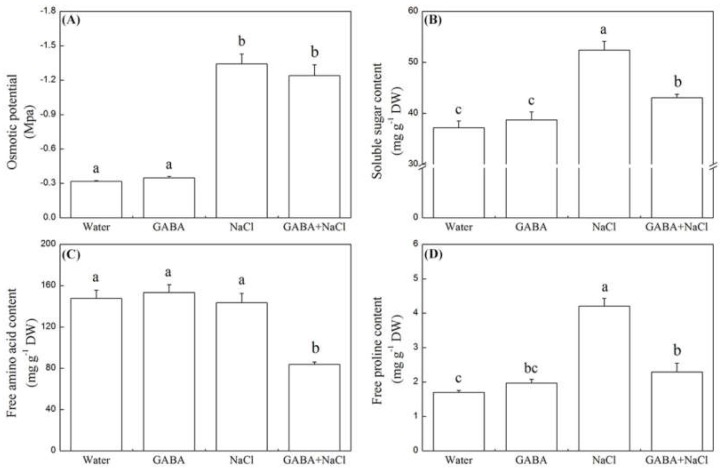
Effects of seed soaking with GABA or water on (**A**) osmotic potential, (**B**) soluble sugar content, (**C**) free amino acid content, and (**D**) free proline content during seeds germination (7 days) under salt stress. Vertical bars indicate ± SE of mean (*n* = 6). Different letters above indicate significant difference. LSD (*p* ≤ 0.05).

Seeds soaking with GABA had no significant impact on starch content and amylase activity under the normal water condition (Figure 2A–D). In response to salt stress, the α-amylase activity was significantly inhibited without GABA pretreatment. However, the seeds soaking with GABA significantly improved total, α-, and β-amylase activity and significantly decreased starch content (Figure 2A–D). There are no significant differences on osmotic potential (OP), soluble sugar, free amino acid, and free proline content between seeds priming with and without GABA under normal water condition (Figure 3A–D). The salt stress significantly increased soluble sugar and free proline content, but decreased OP in seedlings. Seedlings with GABA treatment had distinctly lower soluble sugar, amino acids (AA), and free proline content than seedlings without GABA treatment under salt stress (Figure 3A–D).

**Figure 4:**
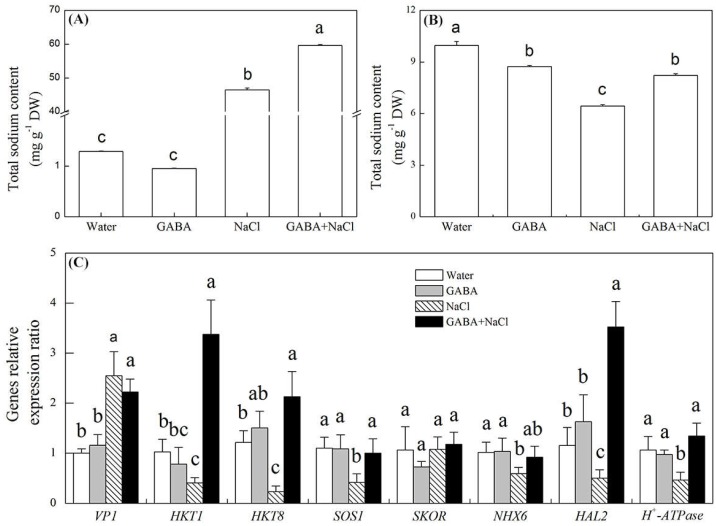
Effects of seed soaking with GABA or water on (**A**) total sodium content, (**B**) total potassium content, and (C) Sodium/potassium transporter gene (*VP1*, *HKT1*, *HKT8*, *SKOR*, *HAL2*, *H^+^-ATPase*, *SOS1*, *NHX6*) relative expression ratio during seeds germination (7 days) under salt stress. Vertical bars indicate ± SE of mean (*n* = 4). Different letters above columns indicate significant difference. LSD (*p* ≤ 0.05).

The GABA treatment did not have an influence on Na^+^ content, but it significantly reduced the K^+^ content under normal water condition (Figure 4A,B). The salt stress significantly increased the Na^+^ content and reduced the K^+^ content (Figure 4A,B). When seeds pretreated with GABA, the Na^+^ and K^+^ content in seedlings significantly increased under salt stress (Figure 4A,B). Under normal water condition, the treatment of seeds soaking with GABA had no effects on transcript levels of genes involved in Na^+^/K^+^ transporter (*VP1*, *HKT1*, *HKT8*, *SKOR*, *HAL2*, *H^+^-ATPase*, *SOS1*, *NHX6*) (Figure 4C). The salt stress significantly improved the *VP1* transcript levels and significantly inhibited transcript levels of *HKT1*, *HKT8*, *HAL2*, *H^+^-ATPase*, *SOS1*, *NHX6* in seedlings without GABA treatment, but did not inhibit these genes expression in seedlings with GABA treatment (Figure 4C). Under salt stress, transcript levels of *HKT1*, *HKT8*, *HAL2*, *H^+^-ATPase* and *SOS1* in seeds soaking with GABA is 8.28, 9.57, 7.04, 2.90, and 2.40 times higher than that in seeds soaking with water (Figure 4C).

### 2.4. Effects of the GABA on Antioxidant Defense and Oxidative Damage

**Figure 5:**
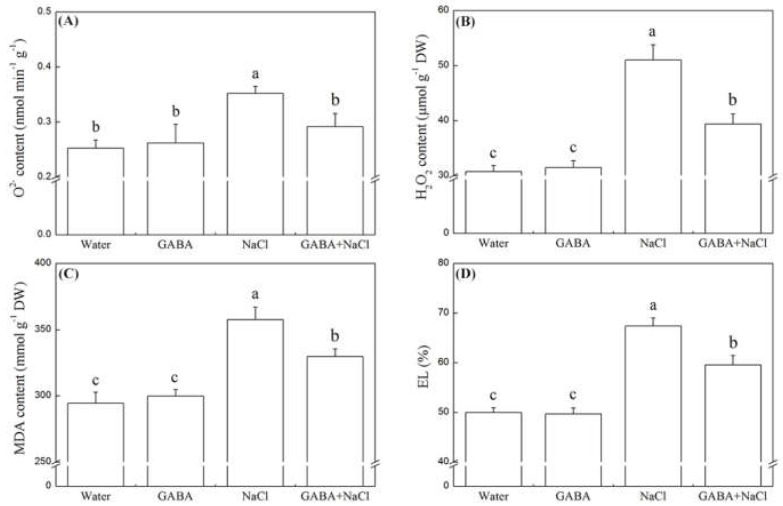
Effects of seed soaking with GABA or water on (**A**) superoxide anion (O_2_^−^) content, (**B**) perhydrol (H_2_O_2_) content, (**C**) malondialdehyde (MDA) content, and (**D**) electrolyte leakage (EL) during seeds germination (7 days) under salt stress. Vertical bars indicate ± SE of mean (*n* = 6). Different letters above indicate significant difference. LSD (*p* ≤ 0.05).

**Figure 6:**
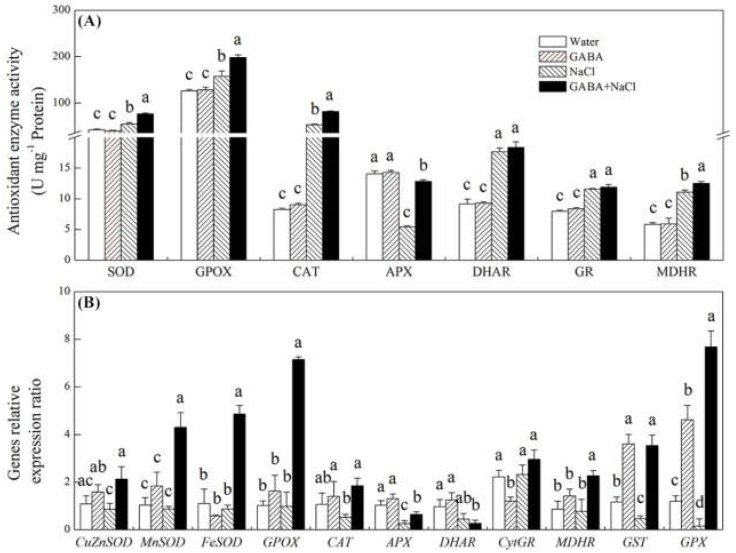
Effects of seed soaking with GABA or water on (**A**) antioxidant enzyme activity and (**B**) gene relative expression ratio during seeds germination (7 days) under salt stress. SOD, superoxide dismutase activity; CAT, catalase activity; GPOX, guaiacol peroxidase activity; APX, ascorbate peroxidase activity; DHAR, dehydroascorbate reductase activity; GR, glutathione reductase activity; MDHR, monodehydroascorbate reductase activity. *Cu/ZnSOD*, *FeSOD* and *MnSOD*, superoxide dismutase genes; *CAT*, catalase gene; *GPOX*, guaiacol peroxidase gene; *APX*, ascorbate peroxidase gene; *DHAR*, dehydroreductase gene; *CytGR*, glutathione reductase gene; *MDHR*, monodehydroascorbate reductase gene; *GPX*, glutathione peroxidase gene; *GST*, glutathione *S*-transferase gene. V*e*rtical bars indicate ± SE of mean (*n* = 4). Different letters above columns indicate significant difference. LSD (*p* ≤ 0.05).

The superoxide anion (O_2_^−^), perhydrol (H_2_O_2_), malondialdehyde (MDA) content, and electrolyte leakage (EL) did not show significant differences between GABA-treated or water-treated seedlings under normal water condition (Figure 5A–D). The salt stress obviously raised O_2_^−^, H_2_O_2_, MDA content, and EL, but seeds soaking with GABA significantly declined O_2_^−^, H_2_O_2_, MDA content, and EL (Figure 5A–D). All detected antioxidant enzyme activities did not show significant differences between seedlings with and without the GABA priming under normal water condition (Figure 6A). The salt stress evidently improved superoxide dismutase (SOD), guaiacol peroxidase (GPOX), catalase (CAT), dehydroascorbate reductase (DHAR), glutathione reductase (GR), and monodehydroascorbate reductase (MDHR) activities, but remarkably inhibited ascorbate peroxidase (APX) activity (Figure 6A). The treatment of GABA significantly improved SOD, GPOX, CAT, APX and MDHR activities under salt stress (Figure 6A). Under normal water condition, exogenous GABA only up-regulated glutathione *S*-transferase (*GST*) and glutathione peroxidase (*GPX*) gene expression (Figure 6B). The salt stress significantly decreased transcript levels of ascorbate peroxidase gene (*APX*)*, GST*, and *GPX* (Figure 5B). Seeds soaking with GABA evidently improved transcript levels of superoxide dismutase genes (*Cu/ZnSOD, MnSOD, FeSOD*), guaiacol peroxidase gene (*GPOX*)**, catalase gene (*CAT*)*, APX*, monodehydro ascorbate reductase gene (*MDHR*)*, GST* and *GPX* under salt stress. The transcript levels of *Cu/ZnSOD, MnSOD, FeSOD*, *GPOX*, *CAT*, *MDHR, GST* and *GPX* in seedlings with GABA treatment is 2.47, 5.81, 5.58, 10.83, 3.54, 2.94, 7.54, and 47.56 times higher than that in seedlings without GABA treatment under salt stress, respectively (Figure 6B).

### 2.5. Effects of the GABA on Accumulation of Dehydrins and Genes Relative Expression

**Figure 7:**
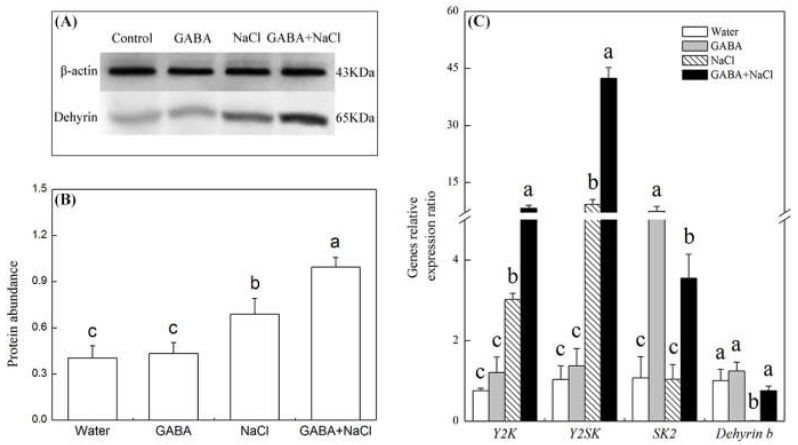
Effects of seed soaking with GABA or water on (**A**) and (**B**) dehydrins abundance and (**C**) dehydrins genes (*SK2*, *Y2K*, *Y2SK* and *dehydrin b*) relative expression ratio during seeds germination (7 days) under salt stress. Vertical bars indicate ± SE of mean (*n* = 4). Different letters above columns indicate significant difference. LSD (*p* ≤ 0.05).

**Figure 8:**
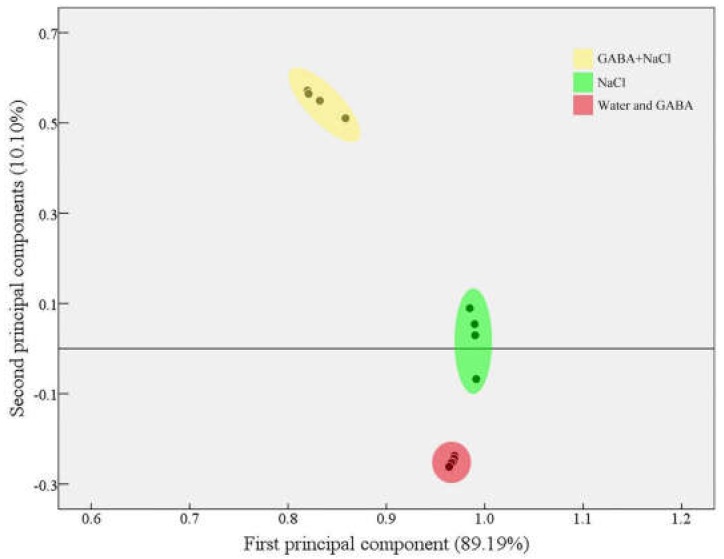
Principal component analysis (PCA) based on analyzed parameters. Each dot indicates each replicate of each treatment. Four treatments were showed in PCA including “Water”, “GABA”, “NaCl”, and “GABA+NaCl”.

Seeds soaking with GABA had no influence on the abundance of dehydrins (65 KDa) during germination under non-stress condition. The salt stress significantly increased the abundance of dehydrins, and the GABA treatment further enhanced the accumulation of dehydrins during germination under salt stress (Figure 7A,B). Under normal water condition, GABA had no significant influence on transcript levels of dehydrins genes (*SK2, Y2K, Y2SK* and *dehydrin b*), but evidently improved *SK2* expression (Figure 7C). The salt stress significantly improved *Y2K* and *Y2SK* transcript levels, but inhibited *dehydrin b* expression (Figure 7C). During germination, seeds soaking with GABA exhibited significantly higher *SK2*, *Y2K*, *Y2SK* and *dehydrin* *b* transcript levels than seeds soaking with water under salt stress. Transcript levels of *SK2, Y2K, Y2SK*, or *dehydrin b* in seedlings with GABA treatment is 2.71, 4.58, 3.42, or 142.19 times as high as transcript levels of these genes in seedlings without GABA treatment under salt stress, respectively (Figure 7C). Principal component analysis (PCA) showed that two principal components explained and predicted 99.29% of the total variance (Figure 8). A distinct separation was obtained among “Water and GABA”, “NaCl”, and “GABA+NaCl”, which indicates that GABA had significant effects on seed germination under salt stress (Figure 8).

## 3. Discussion

The high concentration of salt limits the growth and yield of many crops including white clover [23,24,25,26]. The study of Barbagallo et al. showed that salt stress significantly reduced the GP, GV, and VI of canola seeds [9]. During seeds germination, the salt stress also had obviously negative effects on SL, RL, FW, and DW leading to declines in growth and yield of canola [9]. It has been found that the exogenous GABA could significantly improve the SL, RL, and FW of maize (*Zea mays* L.) seedling under salt stress [27]. In our study, the 100 mM NaCl-induced salt stress made the germination percentage of white clover seeds drop to half of normal germination percentage, indicating that seeds germination of white clover was highly sensitive to salt. Seeds soaking with lower concentration of GABA (0.25, 0.5, and 1 μM) effectively alleviated salt-caused inhibition of germination characteristics and 1 μM of GABA exhibited best effects on the improvement of GP, GV, GI, and VI. In addition, seeds soaking with 1 μM of GABA could also significantly increase SL, RL, FW, and DW of seedlings under salt stress. However, higher concentration of GABA (5 μM) inhibited seeds germination of white clover under salt stress. These results suggest that GABA could significantly improve salt tolerance of white clover seeds, but this effect is dependent on appropriate concentration of GABA.

During seeds germination, the starch catabolism is a primary importance because the process provides available carbohydrates for seeds germination and growth. Previous studies have found that environmental stresses such as drought and salt decreased seeds germination associated with the inhibition of starch catabolism [28,29]. Metabolites of starch catabolism, such as soluble sugar, are also critical for the maintenance of cell turgor and energy sources when seeds are subjected to osmotic and ionic stress [29]. Our results show that the salt stress significantly reduced the starch catabolism and utilization of carbohydrate in seeds soaking with H_2_O, but accelerated starch catabolism through activating α- and β-amylase activities in GABA-pretreated seeds. Interestingly, although seeds soaking with H_2_O had significantly higher organic osmolytes (sugar and proline) than seeds soaking with GABA during germination, salt stress decreased osmotic potential of these two treatments to the same level. As compared to untreated seeds, GABA pretreatment improved Na^+^/K^+^ transportation and the accumulation of Na^+^/K^+^ during germination under salt stress. It is well-known that both organic and inorganic osmolytes are important osmotic regulators for plants adaption to harsh environmental stress [30,31,32]. Our earlier study also found that low concentration of exogenous Na^+^ (30 mM) pretreatment could significantly enhance osmotic adjustment of white clover associated with increases in the Na^+^ absorption and transportation instead of accumulating more carbohydrates and proline [22]. Previous studies have found that Na^+^ sequestration in the vacuole could act as a cheaper osmoregulatory solutes than organic osmolytes because the synthesis and accumulation of organic osmolytes costs more energy in plant cells [33,34]. Our current findings indicated that the increase in starch catabolism induced by GABA mainly provide available carbohydrates for seeds germination and growth of white clover instead of going to osmotic adjustment under salt stress. The maintenance of osmotic potential might depend on the accumulation and transport of Na^+^/K^+^ in seeds soaking with GABA in response to salt stress.

GABA exhibits the important function of ROS scavenging in plants [17]. It has been proved that exogenous GABA could significantly improve multiple antioxidant enzyme activities (GPX, SOD, POD, CAT, APX and GR) in rice (*Oryza sativa*) [35], black pepper (*Piper nigrum*) seedlings [18], and perennial ryegrass (*Lolium* *perenne*) [36], thereby effectively alleviating drought- or heat-caused oxidative damage. Exogenous GABA has also been shown to reduce the low light stress damage through regulating the antioxidant defense system [15]. More importantly, seed soaking with exogenous GABA significantly improved SOD, POD, and CAT activities in roots and leaves of tomato seedlings associated with declines in the production of ROS and oxidative damage under NaCl stress [21]. In the current study, we found that exogenous GABA significantly improved the activities of SOD, POD, CAT, APX, and MDHR in seedlings of white clover under salt stress. Those GABA-activated antioxidant enzymes could play key roles in scavenging ROS such as H_2_O_2_ and O_2_^∙−^ and reducing membrane lipid peroxidation during seeds germination under salt stress. The study of Shi et al. also found that exogenous GABA could inhibit key genes expression of H_2_O_2_ synthesis to reduce the accumulation of H_2_O_2_ in *Caragana* *intermedia* roots, suggesting that GABA might act as a signal molecule to regulate the gene expression in response to salt stress [14]. Our findings also suggested that GABA mediated antioxidant defense in white clover during seeds germination at the molecular level through up-regulating multiple genes (*Cu/ZnSOD*, *MnSOD*, *FeSOD*, *GPOX*, *CAT*, *APX*, *MDHAR*, *GST*, and *GPX*) encoding antioxidant enzymes in response to salt stress.

Dehydrins (late embryogenesis abundant protein) play fundamental roles in plant adaptation to abiotic stress. There are almost no dehydrins in the normal vegetative tissue, but dehydrins largely accumulate during seeds germination or under salt, dehydration, freezing, and heat stress in vegetative tissues [37,38]. A large number of transgenic studies revealed positive effects of dehydrins accumulation or the expression of genes encoding dehydrins on stress tolerance in different plant species [37]. The study of Ruibal et al. found that specific dehydrins (PpDHNA, PpDHNB, DHNA and DHNB) could improve salt and drought tolerance of *Physcomitrella patens* [39]. The study of Hundertmark et al. found that different types of dehydrins (LEA14, XERO1 and RAB18) could protect seeds against deterioration during low moisture storage and increase seeds germination of *Arabidopsis thaliana* under salt stress [40]. It has been proved that exogenous plant growth regulators such as ABA, cytokinin, PAs, and proline can induce dehydrins accumulation or the expression of genes encoding dehydrins associated with the improvement of stress tolerance in various plants species including white clover [41,42,43]. The study of Li et al. has found that Spm could significantly promote the accumulation of dehydrins in white clover under osmotic stress, thereby improving the drought tolerance of white clover [44]. In the current study, we found that exogenous GABA significantly increased the accumulation of dehydrins (65 kDa) and expression levels of four genes encoding dehydrins (*SK2, Y2K, Y2SK*, and *dehydrin b*) during seeds germination under salt stress, indicating GABA-regulated seeds germination and stress tolerance were closely related to dehydrins accumulation in white clover in response to salt stress.

## 4. Materials and Methods

### 4.1. Plant Materials and Treatments

Seeds of white clover (cv. Haifa) were surface-sterilized for 5 min in 0.1% HgCl and then rinsed four times with distilled water (ddH_2_O). For soaking pretreatment, one set of seeds was soaked in ddH_2_O for 3 h as control and another set of seeds was soaked in ddH_2_O for 1 h and then soaked in different concentrations of GABA for 2 h at 20 °C, respectively. The soaked seeds were then germinated in petri dishes (a diameter of 90 mm) with four sheets of filter paper containing 10 mL of 0 or 100 mg·L^−1^ NaCl. Each treatment was replicated six times (50 seeds for each duplicate). The petri dishes were kept in a growth chamber programmed at average day/night temperature of 23/19 °C, 75% relative humidity, and 700 μmol·m^−2^·s^−1^ photosynthetic photon flux density for 7 days. The ddH_2_O was added in each petri dish every day until the weight of each Petri dish reached its initial weight. Seeds were sampled at 7 d of germination for biochemical and physiological measurements.

### 4.2. Determination of Seed Germination Characteristics, Root Viability and Endogenous GABA Content

GV was evaluated after 3 d of germination, and GP was evaluated after 7 days of germination. GI and MGT were calculated based on the following formula:

(1) $$GI=\sum \frac{Gt}{Tt}$$

where *Gt* is the number of the germinated seeds in the *t* day; *Tt* is the time corresponding to *Gt*:

(2) $$MGT=\frac{\sum Ti\times Ni}{\sum Ni}$$

where *Ni* is the number of the new germination seeds in times of *Ti*, respectively [10]. 

After 7 d of germination, RL, SL, shoot-root ratio, FW, DW, and VI were calculated. VI was measured based on the formula VI = FW × GI [29]. For root viability, fresh roots (0.1 g) were randomly sampled, and then the fresh roots, 0.4% TTC (1 mL), and 0.0667 M phosphate buffer (1 mL, pH 7.4) were added to the pellet and incubated at 37 °C for 1 h in the dark. The sulfuric acid (1 M, 2 mL) was added in order to stop reaction. After moving roots into a new pellet, methanol (5 mL) was added and roots were incubated at 40 °C for 7 h. The absorbance of the supernatant was measured at 485 nm [45]. For the determination of endogenous GABA content, the method of enzyme linked immunosorbent assay (ELISA) was used. The assay kit was purchased from Shanghai Enzyme-linked Biotechnology Co., Ltd., China. Briefly, 0.1 g of seedlings were grounded in 1 mL of 0.1 M PBS (pH 7.4) and then centrifuged at 4 °C for 15 min. The supernatant was used for measurement of endogenous GABA content. The procedure was carried out according to the specification and the absorbance was read at 450 nm after adding stop solution within 15 min on a microplate reader (Synergy HTX, Bio Tek, Winooski, VT USA).

### 4.3. Determination of Starch Metabolism, Amino Acids, and Osmotic Potential

For water soluble carbohydrate quantification, the procedure was conducted following the method of Fu and Dernoeden [46]. Seedlings (0.5 g) were collected and dried in an oven. Dry tissue (0.05 g) was placed in 10 mL centrifuge tube, and 6 mL ethanol (80%) was added. The mixture was extracted in the water bath at 80 °C for 30 min and then centrifuged at 12,000 *g* for 10 min. The supernatant was used to measure the content of water-soluble carbohydrate (WSC) [44], and the residue was obtained for starch content analysis [47]. The activities of amylase enzymes were measured by using the method of Tarrago and Kishorekumar et al. [48,49]. Seedlings (0.1 g) were ground with distilled water (8 mL) at 4 °C. The extract was centrifuged at 12,000 *g* for 25 min at 4 °C. The supernatant was used for estimating α- and β-amylase activities. The 3 mL of supernatant mixed with 3 mL of CaCl_2_ (3 mM) and incubated at 70 °C for 5 min. The reaction mixture (0.1 mM citrate buffer, 2% soluble starch solution, and 0.7 mL hot enzyme extract) was incubated at 30 °C for 6 min and then the mixture was heated for 5 min at 50 °C. The α-amylase activity was estimated spectrophotometrically at 540 nm. After inactivating α-amylase at pH 3.4, the β-amylase activity was determined. Reaction solution (2 mL of 0.1 mM citrate buffer, 2% soluble starch, and 0.7 mL EDTA treated enzyme extract) was incubated at 30 °C for 5 min after the addition of starch. The β-amylase activity was then assayed as same as α-amylase.

OP in seedlings was measured according to the method of Blum [50]. Collected seedlings were immediately frozen in liquid nitrogen for 10 min. Seedlings were thawed for 25 min at 4 °C, and then pressed the cell sap for determination of the osmolarity (c) using a sampling chamber of osmometer (Wescor, Logan, UT, USA). The OP was converted based on the formula: MPa = −c × 2.58 × 10^−3^. Free amino acid content was estimated by the spectrophotometric method using a microplate reader (Synergy HTX, Bio Tek, USA). The assay kit was purchased from Suzhou Comin Biotechnology Co., Ltd., China. Free proline content in seedlings was estimated according to Bates et al. method [51]. 0.1 g of the seedlings sample were weighed and homogenized into a fine paste in 10 mL of 35% sulphosalicylic acid. The homogenate was centrifuged for 10 min. 2 mL of supernatant was mixed with 2 mL of glacial acetic acid and 2 mL of acid ninhydrin solution. The mixture was heated on a water bath for 1 h and the reaction was terminated by keeping in the ice-cold conditions. 4 mL of toluene was added, and the absorbance was read at 520 nm. 

### 4.4. Determination of Antioxidant Enzyme Activities and Oxidative Damage

To analyze antioxidant enzyme activities, fresh seedlings (0.2 g) were ground with 50 mM cold phosphate buffer (4 mL, pH 7.8) containing 1% (*w*/*v*) polyvinylpyrrolidone. The homogenate was centrifuged at 12,000 *g* for 30 min at 4 °C. The supernatant was used for assays of antioxidant enzyme activities and MDA content. The SOD activity was measured by recording the rate of p-nitroblue tetrazolium chloride reduction of the absorbance at 560 nm [52]. The activity of CAT, GPOX, APX, MDHR, DHAR and GR was determined by following the changes in absorbance at 240, 470, 290, 340, 265 and 340 nm, respectively [53]. Protein content was determined using Bradford’s method [54]. The content of MDA was measured using the method of Dhindsa et al. [55]. Briefly, enzyme extract (0.5 mL) and reaction solution (1 mL) containing 20% (*w*/*v*) trichloroacetic acid and 0.5% (*w*/*v*) thiobarbituric acid were added to the pellet. The mixture was heated in a water bath at 95 °C for 15 min, and then cooled quickly in an ice-water bath. The homogenate was centrifuged at 8000 *g* for 10 min. The absorbance of the supernatant was measured at 532, 600 and 450 nm. 

The formation rate of O_2_^∙−^ was measured using the sulfanilamide method and the absorbance was measured at 530 nm [56]. H_2_O_2_ was assayed according to the method of potassium iodide. The oxidation product was measured at 390 nm [57]. For electrolyte leakage (EL), fresh seedlings (0.1 g) were immersed in the centrifuge tube with deionized water (15 mL). The tubes were shaken for 24 h on a shaker table. The conductivity of the solution (C_initial_) was measured using a conductivity meter (DDS-307A, Shanghai Precision and. Scientific Instrument Co., Ltd., Shanghai, China). Seedlings were then killed by autoclaving at 140 °C for 30 min. The conductivity of killed tissues (C_max_) was measured. Relative EL was calculated as the percentage of C_initial_ over C_max_ [58].

### 4.5. Determination of Na^+^/K^+^ Content and Western Blot Analysis 

For Na^+^/K^+^ content, seedlings (7 day) were dried at 105 °C for 2 h and then maintained at 75 °C for 72 h. Dry tissue (0.3 g) was added in10 mL of concentrated sulfuric acid, and the mixture was put in graphite digestion instrument (Hanon-SH220N/SH220F, Hanon Subsidiary Company, Shanghai, China). The sample was dissolved completely, and the sample was measured by a flame photometer (Inesa-FP6413, INESA, Shanghai, China). For the analysis of western blot, soluble proteins were extracted from 0.5 g seedlings in ice cold 100 mM Tris-HCl buffer (pH 8.0) and then centrifuged at 12,000 *g* for 10 min (4 °C). The supernatant was collected and boiled for 10 min. After recentrifuging at 12,000 g, the sediment (an equal amount of 30 μg proteins) was used for determination of dehydrins. The Bio-Rad mini protean transblotter was used for transferring SDS-PAGE (12%) to PVDF membranes. After 2 h of transference at 4 °C and 65 V, the membranes were blocked in TRIS-buffered saline for 1 h [59,60]. When the TRIS-buffered saline was removed, the membranes were washed briefly in TTBS for 3 times each for 5 min. The washed membranes were incubated in rabbit anti-dehydrins dilution (1:1000) for 1 h. After that, the membranes were rinsed in TTBS for 3 times each for 5 min again and incubated in dilution of goat anti-rabbit IgG antibody (1:2000) as the second antibody for 1 h. After washing in TTBS for 20 min, the dehydrins bands were detected by using the TMB reagent kit (Sigma, Kawasaki, Japan) [61].

### 4.6. Genes Expression Analysis

Transcript levels of genes were performed using a real-time quantitative polymerase chain reaction (qRT-PCR). For total RNA, the 0.1 g of fresh seedlings was extracted by using RNeasy Mini Kit (Qiagen) according to instructions. A revert Aid First Stand cDNA Synthesis Kit (Fermentas) was used for reverse-transcribing RNA to cDNA. The cDNA was subjected to qPCR using primers of antioxidant enzyme genes (*Cu/ZnSOD, FeSOD, MnSOD, CAT, GPOX, APX, MDHR, DHAR, GPX, CytGR, GST*) [29], dehydrin genes (*Y2SK, Y2K, SK2*) [60], and Na^+^/K^+^ transporter genes (*VP1, HKT1, HKT8, SKOR, HAL2, H^+^-ATPase, SOS1, NHX6*) (Table 3) [22]. Transcript level of each gene was measured using an iCycleriQqRT-PCR detec-tion system with SYBR Green Supermix (Bio-Rad). Four biological replicates with independent cDNA preparations were tested in this study. The conditions of the PCR protocol for all genes were as follows: 5 min at 94 °C and denaturation at 95 °C for 30 s (40 repeats), annealing at 57–66 °C (Table 3) for 30 s and extension at 72 °C for 30 s. At the end of PCR cycle, the transcript level of all genes was calculated according to the formula 2^−ΔΔ*C*t^ described by Xia et al. [62].

### 4.7. Statistical Analysis

The data was analyzed by using SPSS 20 (IBM, Armonk, NY, USA). The significant relationships among the treatments are tested based on differences between means at *p* ≤ 0.05.

## 5. Conclusions

Seeds priming with the appropriate concentration of GABA could be an effective technique to alleviate salt-caused inhibition of seeds germination. During germination, seeds soaking with GABA accelerated starch metabolism and utilization of soluble sugar and amino acids contributing to the maintenance of better growth than seeds soaking with H_2_O under salt stress. In response to salt stress, GABA-induced increases in Na^+^/K^+^ accumulation and transportation could play important roles in osmotic adjustment through offsetting the overconsumption of organic osmolytes during seeds germination. GABA could also increase antioxidant enzyme activities and genes transcript levels associated with better maintenance of cell membrane stability in seedlings of white clover under salt stress. In addition, GABA further increased the accumulation of dehydrins in salt-stressed seedlings, which could be another important regulatory mechanism of salt tolerance in white clover. Current findings provide new evidence for better understanding of GABA-regulated salt tolerance during seeds germination.

## Figures and Tables

**Table 1:** Effects of seed priming with water or γ-aminobutyric acid (GABA) on seed germination characteristics in white clover under seven days of different salt stress conditions. Values are mean ± standard error (SE) (n = 6). Different letters in a vertical column indicate a significant difference between each treatment under different NaCl concentration. The asterisk (*) indicates a significant difference exists between seed priming with water or GABA. LSD (*p* ≤ 0.05).

GABA (µM)	Germination Percentage (%)		Germination Vigor (%)		Germination Index		Mean Germination Time (d)		Seed Vigour Index	
	Water	NaCl	Water	NaCl	Water	NaCl	Water	NaCl	Water	NaCl
0.00	95.33 ± 1.15 ^a^	51.50 ± 1.00 ^cd^*	90.80 ± 1.10 ^a^	33.33 ± 2.31 ^b^*	35.66 ± 1.28 ^a^	9.29 ± 0.75 ^c^*	1.61 ± 0.05 ^a^	3.28 ± 0.29 ^a^*	1.58 ± 0.13 ^a^	0.34 ± 0.02 ^d^*
0.25	96.00 ± 1.00 ^a^	54.67 ± 1.15 ^bc^*	93.20 ± 1.10 ^a^	40.67 ± 1.15 ^a^*	35.66 ± 1.47 ^a^	10.46 ± 0.16 ^b^*	1.63 ± 0.02 ^a^	3.10 ± 0.21 ^a^*	1.59 ± 0.04 ^a^	0.38 ± 0.01 ^c^*
0.50	95.60 ± 1.67 ^a^	58.00 ± 5.29 ^ab^*	93.33 ± 1.15 ^a^	39.33 ± 3.06 ^a^*	35.86 ± 1.97 ^a^	11.05 ± 0.88 ^ab^*	1.85 ± 0.52 ^a^	3.07 ± 0.14 ^a^*	1.59 ± 0.09 ^a^	0.41 ± 0.03 ^b^*
1.00	96.00 ± 1.41 ^a^	63.33 ± 4.16 ^a^*	93.50 ± 1.00 ^a^	42.00 ± 2.00 ^a^*	34.87 ± 3.00 ^a^	11.73 ± 0.11 ^a^*	1.75 ± 0.15 ^a^	3.06 ± 0.12 ^a^*	1.53 ± 0.19 ^a^	0.45 ± 0.01 ^a^*
2.50	96.00 ± 2.83 ^a^	50.50 ± 4.43 ^d^*	92.50 ± 1.91 ^a^	32.67 ± 1.15 ^b^*	33.92 ± 1.57 ^a^	9.15 ± 0.29 ^c^*	1.75 ± 0.09 ^a^	3.07 ± 0.09 ^a^*	1.48 ± 0.09 ^a^	0.35 ± 0.01 ^d^*
5.00	95.60 ± 0.90 ^a^	46.00 ± 4.90 ^d^*	92.50 ± 1.91 ^a^	32.00 ± 3.46 ^b^*	34.19 ± 2.15 ^a^	8.77 ± 0.26 ^c^*	1.68 ± 0.06 ^a^	3.12 ± 0.19 ^a^*	1.51 ± 0.11 ^a^	0.32 ± 0.01 ^d^*

**Table 2:** Effects of seed priming with water or GABA on seed germination characteristics in white clover under seven days of different salt stress conditions. Values are mean ± SE (n = 6). Different letters in a vertical column indicate a significant difference between each treatment under different NaCl concentration. The asterisk (*) indicates a significant difference exists between seed priming with water or GABA. LSD (*p* ≤ 0.05).

GABA (µM)	Seedling Fresh Weight (mg·10 Seedling^−1^)		Seedling Dry Weight (mg·10 Seedling^−1^)		Root Length (cm)		Shoot Length (cm)		Shoot-Root Ratio	
	Water	NaCl	Water	NaCl	Water	NaCl	Water	NaCl	Water	NaCl
0.00	44.26 ± 2.44 ^a^	36.25 ± 0.96 ^c^*	3.36 ± 0.15 ^a^	3.08 ± 0.2 ^bc^*	1.03 ± 0.09 ^a^	0.21 ± 0.02 ^c^*	0.35 ± 0.03 ^a^	0.25 ± 0.01 ^b^*	2.85 ± 0.23 ^a^	0.85 ± 0.11 ^b^*
0.25	45.38 ± 0.88 ^a^	36.30 ± 0.70 ^bc^*	3.33 ± 0.21 ^a^	3.18 ± 0.27 ^ab^	1.07 ± 0.10 ^a^	0.24 ± 0.01 ^bc^*	0.37 ± 0.02 ^a^	0.27 ± 0.02 ^b^*	2.80 ± 0.30 ^a^	0.87 ± 0.06 ^b^*
0.50	45.58 ± 3.24 ^a^	37.33 ± 0.60 ^ab^*	3.30 ± 0.20 ^a^	3.13 ± 0.12 ^ab^	1.10 ± 0.06 ^a^	0.23 ± 0.01 ^bc^*	0.36 ± 0.02 ^a^	0.27 ± 0.01 ^b^*	2.98 ± 0.08 ^a^	0.87 ± 0.09 ^b^*
1.00	44.82 ± 1.78 ^a^	37.97 ± 0.87 ^a^*	3.35 ± 0.10 ^a^	3.45 ± 0.13 ^a^	1.06 ± 0.06 ^a^	0.29 ± 0.04 ^a^*	0.37 ± 0.02 ^a^	0.30 ± 0.02 ^a^*	2.85 ± 0.17 ^a^	0.98 ± 0.10 ^a^*
2.50	42.68 ± 0.81 ^a^	37.77 ± 0.59 ^a^*	3.33 ± 0.12 ^a^	3.15 ± 0.26 ^ab^	1.00 ± 0.01 ^a^	0.26 ± 0.04 ^ab^*	0.37 ± 0.02 ^a^	0.26 ± 0.02 ^b^*	2.77 ± 0.12 ^a^	0.87 ± 0.11 ^b^*
5.00	42.80 ± 1.77 ^a^	36.07 ± 0.58 ^c^*	3.26 ± 0.06 ^a^	2.83 ± 0.21 ^c^*	1.01 ± 0.03 ^a^	0.21 ± 0.02 ^c^*	0.37 ± 0.01 ^a^	0.26 ± 0.01 ^b^*	2.88 ± 0.30 ^a^	0.76 ± 0.05 ^b^*

**Table 3:** Primer sequences and their corresponding GeneBank accession numbers of the analyzed genes.

Targetgene	Accession No.	Forward Primer (5′–3′)	Reverse Primer (5′–3′)	Tm (°C)
*Cu/ZnSOD*	JQ321597.1	AACTGTGTACCACGAGGACTTC	AGACTAACAGGTGCTAACAACG	58
*FeSOD*	KP202173	ACACGATTTCTCAGGGTTACGAC	GCGGCCAAGACTATCAGTTCCAT	58
*MnSOD*	JQ321598.1	TAAGGGAACCTACCCGATAACT	CCAGGACCAAACGTCACCAAAG	66
*CAT*	JQ321596.1	AACAGGACGGGAATAGCACG	ACCAGGTTCAGACACGGAGACA	58
*GPOX*	JQ321606.1	CACTTGGTTTAGTTTTGTCGCC	AACACGGTCTTGTCTGCTACG	64
*APX*	JQ321599.1	TAAAGATAGTCAACCCACCTCAACA	ACCAGTCTTGGGAAACAACGTA	58
*MDHR*	KP202172	CCAACTGCCTAAAGCCACATCT	GAAGAAAGGAAACTAACGGAGCAT	64
*DHAR*	KP202171	TGGTTACCTCCCGACCCTAT	TCTTACCAAGGAACTTTAGTCAGG	58
*GPX*	JQ321604.1	ATGTGCCTTGAGAGCGTGAATATAC	CCTTTAAGACGAAACTTGGACC	58
*CytGR*	JQ321602.1	TAAACTTCCACTCCCTTTCTATCG	CTACAATATGGGTTGAGGACAGGT	58
*GST*	JQ321600.1	TCGAGCTAAGACCCGACTAATAC	GAGGTTGTTAAACTACCGAAGATAC	58
*SK2*	GU443960.1	TGGAACAGGAGTAACAACAGGTGGA	TGCCAGTTGAGAAAGTTGAGGTTGT	58
*Y2K*	JF748410.1	AGCCACGCAACAAGGTTCTAA	TTGAGGATACGGGATGGGTG	60
*Y2SK*	GU443965.1	GTGCGATGGAGATGCTGTTTG	CCTAATCCAACTTCAGGTTCAGC	60
*dehydrin b*	GU443960.1	TCCAGTCATCCAGCCTGTTG	CCAGCCACAACACTTGTCA	60
*VP1*	MF405364	GTCCAATCAGTGACAATGCCG	AGAGGGCAAGAGACACAAGAGC	58
*HKT1*	MF405365	TGCATCACCGAAAGACAAAGC	ATCGACAACCCTACATTCCCATA	57
*HKT8*	MF405366	TTCAAGACACGCTGGAGAAACTAT	CGATGGCAGGAATGAGGTGT	57
*SKOR*	MF405367	GTTTCATTTGATATGGTTCTCGGTG	GGCCCTTTATTTGTTCACGGA	58
*HAL2*	MF405368	TTGTGAACCAGTTGAGAAGGCC	TCGGCATCTCCACGACCTATT	61
*H^+^–ATPase*	MF405369	CGTATAGTGTTTGGCTTCATGTTCA	AATGGAGATGGCACCACCCTA	60
*SOS1*	MF405370	TGGTCCATCTGAAAGTGACAATAAC	TCATCAAGCATCTCCCAGTAAGC	57
*NHX6*	MF405371	CAGTCTGGTTTCAGTCTTGCTCC	ACCAAACATCAGGCACTCAACA	60
